# Burden among caregivers of people with mental illness at Jimma University Medical Center, Southwest Ethiopia: a cross-sectional study

**DOI:** 10.1186/s12991-019-0233-7

**Published:** 2019-06-24

**Authors:** Mohammed Ayalew, Abdulhalik Workicho, Elias Tesfaye, Hailemariam Hailesilasie, Mubarek Abera

**Affiliations:** 1 Department of Psychiatry Nursing, School of Nursing, College of Medicine and Health Sciences, Hawassa University, Hawassa, Ethiopia; 2 Department of Epidemiology, Institute of Health, Jimma University, Jimma, Ethiopia; 3 Department of Psychiatry, Institute of Health, Jimma University, Jimma, Ethiopia

**Keywords:** Burden, Caregiver, Mental illness, Jimma, Ethiopia, Africa

## Abstract

**Background:**

Burden of caregivers of people with mental illness (PWMI) is considered to be a negative impact of the care provided by the family to the patient. However, little is known about the extent of the burden among caregivers of PWMI in Ethiopia. The aim of this study, therefore, is to assess the magnitude and associated factors of burden among caregivers of PWMI at Jimma University Medical Center, 2017.

**Methods:**

Institution-based cross-sectional study design was employed among 406 conveniently selected caregivers of PWMI and interviewed using a structured questionnaire. Family burden interview schedule (FBIS) was used to assess burden of caregivers. Bivariate and multivariable linear regression analyses were performed to determine the predictors of burden among caregivers.

**Results:**

Nearly two-thirds [264 (65.0%)] of the participants were male with a mean age of 38.45 ± 12.03 years. The mean score for burden among caregivers on family burden interview schedule was 23.00 ± 10.71. Age of the caregivers (*β* = 0.18, *p* < 0.001), being female caregiver (*β* = 2.68, *p* < 0.01), duration of contact hours with the patient per day (*β* = 0.74, *p* < 0.001), perceived stigma by the caregiver (*β* = 0.47, *p* < 0.001), and providing care for patients who had history of substance use in life (*β* = 1.52, *p* < 0.05) were positive predictors of higher burden among caregivers. Whereas, caregivers’ income (*β* = 7.25, *p* < 0.001), caregivers who had no formal education (*β* = 4.65, *p* < 0.01), and caregivers’ social support (*β* = 0.78, *p* < 0.001) were negatively associated with higher burden among caregiver.

**Conclusion:**

Caregivers of people with mental illness experience enormous burden during providing care for their relatives with mental illness. Therefore, creating community awareness and targeted interventions in the area of treatment access, stigma, financial, and other social support for people with mental illness and their caregivers would help out to reduce these burdens.

## Background

Mental illness is a condition characterized by significant disturbance in cognitive, emotional regulation, and behavioral functioning. Common mental illness includes schizophrenia, depression, bipolar, and anxiety disorders [1]. Mental illness results in an enormous social and economic burden to individuals affected by the illness, their families and communities [2].

Burden of caregiver is any unwanted or negative consequences experienced by caregivers of people with mental illness (PWMI) as a result of taking care of responsibility for PWMI [3, 4]. It can be either objective burden such as family disruption, financial crisis, limitations on activities of daily living and social interactions, and/or subjective burden which is a perceived feeling of getting overwhelmed by the care they are providing [4]. In general, burden among caregivers encompasses physical, psychological, emotional, social, and financial difficulties that family members faced because of taking care of responsibility for PWMI [5].

Studies showed that one in four families has at least one member currently suffering from some sort of mental illness [6] and more than 90% of these PWMI live with and gets continuous support from their families [7]. Caring for PWMI demands considerable amount of time, energy, finance, and other resources from caregivers, and they suffer twice more than the general population [7].

Evidences from developed countries indicated that more than seven in ten (72%) caregivers of PWMI experience significant burden [8]. Nearly, 40% of primary caregivers of people with severe mental health problems experience burden as a result of taking care of responsibility to the patient [9]. Researchers have documented that magnitude of burden among caregivers of PWMI in sub-Saharan countries is high ranging from 60 to 90% across different regions [10–12]. Moreover, the magnitude of burden among caregivers depends on several factors, including the age and sex of the caregivers, pre-morbid relationship between the patient and caregivers, the nature of the patient’s illness, the coping strategies of the caregivers, and cultural and ethnic variables [13].

In Ethiopia, studies showed that nearly two-thirds (63.3%) schizophrenic and bipolar-I disorder patients’ caregivers experience moderate to severe level of burden [14]. Almost all (99%) caregivers who provide care for mentally ill patients stated that they experience moderate to severe level of subjective burden [15].

In low- and middle-income countries (LMICs), the ratio of mental health professionals with PWMI is very low and PWMI have low access to modern mental health services [16]. These imply that the central role of caregiving for mentally ill patients lie on the shoulders of the family members [16]. Because of this, mental illnesses have considerable off-putting impact on the quality of life of patients and their caregivers or friends [17]. Therefore, taking care of responsibility to those with mental illness can affect the dynamics of a family and needs continuous tireless effort, energy, and empathy from caregivers [18].

Therefore, understanding the degree of caregivers’ burden and related factors is crucially significant to plan family intervention programs [19], and also little is known about the extent of the problem in Ethiopia. So, this study will be a benchmark reporting the magnitude and associated factors of burden among caregivers of PWMI in Ethiopia. The hypothesis of this study was that the magnitude of caregiver burden is associated with the socio-demographic and clinical characteristics of the patients and their caregivers and the available social support of caregivers.

## Methods and materials

### Study design and setting

Hospital-based cross-sectional study was conducted at Jimma University Medical Center (JUMC) psychiatric clinic from June 1 to 30, 2017 among caregivers of PWMI. According to the hospital report, currently it is the only teaching and referral hospital in the southwestern part of Ethiopia, providing services for approximately 15,000 inpatient attendants, 160,000 outpatient attendants, 11,000 emergency cases, and 4500 deliveries in a year coming to the hospital from the catchment population of about 15 million people. An average of 750–1000 psychiatric patients has follow-up visit at the psychiatric clinic every month. The psychiatry clinic delivers 24-h emergency service, outpatient regular service, and inpatient/admission services. Currently, the clinic has more than 40 inpatient beds for general adult and child psychiatric patients and substance abuse detoxification treatment.

### Sample size and sampling procedure

Sample size was determined using single population proportion formula. The parameters used to estimate the sample size were as follows: a 60% burden among caregivers of PWMI [12], 5% type I error, 95% confidence level, and non-response rate of 10%. Therefore, the total sample size of the study was 406 caregivers of PWMI. Consecutive sampling technique was used for this study; and family caregivers of mentally ill patients who were visiting psychiatry clinic during the study period, and who fulfilled the inclusion criteria, were included in the study until the final study sample size was reached.

### Inclusion and exclusion criteria

Participants were eligible to participate in the study if they were aged 18 years or above, and are primary caregivers of the patient. Caregivers who were either mentally or physically ill were excluded from the study.

### Measurement

Data were collected using interviewer administered structured questionnaire and patient chart were reviewed to identify patients’ diagnosis and other medical information.

To assess burden of caregivers among PWMI, family burden interview schedule (FBIS) was used. This scale measures objective (24 items) and subjective (1 general standardized question) aspects of burden. It has six subdomains for objective burden and these include financial burden, effects on family routine, effects on family leisure, effects on family interaction, effects on physical health of family members, and effects on mental health of other family members. Each item has three response categories including 0 (no burden), 1 (moderate burden), and 2 (severe burden) and the total scores range from 0 to 48 for objective burden, with higher score indicating a higher burden of care [20]. The cut-off points for FBIS is 0 for no burden, 1–16 for mild burden, 17–32 for moderate burden, and 33–48 for severe burden [21]. One general question to assess subjective burden was “How much would you say you have suffered owing to the patient’s illness?” and will be scored as severely (2), little (1), or not at all (0). Originally in India, the reported reliability and validity score were more than 0.87 and 0.72, respectively [20]. It was validated in different parts of the world. In Germany, reliability for the global objective burden was *α* = 0.83, for the global percentage of subjective burden in *a* = 0.88, and for the entire scale was 0.92 [22]; in Hong Kong, its Cronbach’s alpha for internal consistency was 0.90, intra-class correlation coefficient for inter-rater and test–retest reliability were 0.988 and 0.986, respectively [23]. Also in an African study conducted in Nigeria, the reliability was 0.94 with additional test–retest reliability of 0.83 for the total objective scale score [10]. In this study, its Cronbach’s *α* was 0.939.

The 3-item Oslo Social Support Scale (OSSS) was used to assess social support among caregivers of PWMI [24]. Clinical global impression-severity (CGI-S) scale with 7 points was used to assess the severity of illness [25]. To measure caregivers’ feeling of perceived stigma, a 5-item perceived stigma scale was used; adopted from Verhaeghe and Bracke [8]. The questionnaire was translated from English to local Amharic and Affan-Oromo languages and translated back to English by independent translators to check for semantic validity.

The data were collected by B.Sc. psychiatry nurses and supervised by M.Sc. mental health professionals. Two days of training for data collectors and supervisors were given on the research tool, data collection methods, and how to handle ethical issues. Pre-test was conducted on 5% of the study sample to ensure understandability and applicability of the tool. Each day during data collection, filled questionnaires were cheeked for completeness and consistency by supervisors and principal investigator. Incomplete questionnaires were discarded.

### Data processing and analysis

After checking for the completeness of the questionnaires, data were coded and entered into Epi-Data version 3.1 and exported to SPSS version 20 for analysis. Descriptive statistics like frequencies and percentages were calculated for categorical variables. Mean median, standard deviation, and interquartile range were calculated for continuous variables. Assumptions like presence of normal distributions, lack of multi-collinearity among explanatory variables, presence of linearity relationship, independence and homoscedasticity of the errors were checked. Log-transformation for caregiver income, duration of care provided, and patient age was done to fulfill the assumptions for normality of distribution. Bivariate and multivariate linear regression was conducted to identify independent predictors of the outcome variable. The *p* values of < 0.05 were declared statistically significant.

### Operational definitions

Caregiver: A family member/relative/any person who has most frequent contact with the patient, provides unpaid support to the patient financially, socially, psychologically, and physically, and has mostly been a collateral in the patient’s treatment.

Mental illness: A condition characterized by significant disturbance in cognitive, emotional regulation, and behavioral functioning such as schizophrenia, depression, bipolar, anxiety disorders, etc. and diagnosed according to DSM-5.

## Results

Out of the total 406 caregivers, nearly two-thirds [264 (65.0%)] were male, 275 (67.7%) were married and more than one-fourth [112 (27.6%)] were single. The mean age of the caregivers was 38.45 ± 12.03 years (with a range of 19–65 years) and the median monthly income was 50.00 $US (IQR = 50.00 $US). The median duration of caregiving was 3 (IQR = 6) years. Half [203 (50%)] of the caregivers reside in the rural area, 156 (38.4%) caregivers were caring for their son or daughter and nearly one-third [130 (32.0%)] were caring for their sibling. The details of the descriptive characteristics are presented in Table 1.

**Table 1:** Description of socio-demographic and clinical characteristics of caregivers (*n* = 406)

Variables	Category	*n*	%
Mean age (± SD)		38.45 ± 12.03	
Median income (IQR)		50.00/50.00	
Median duration of care (IQR) in years		3.00/6.00	
Mean duration of stay with patient in 24 h		7.52 ± 3.25	
Mean social support (OSSS) ± (SD)		8.14 ± 3.38	
Mean stigma (PSS) ± (SD)		14.02 ± 6.81	
Sex	Male	264	65.0
	Female	142	35.0
Marital status	Single	112	27.6
	Married	275	67.7
	Others	19	4.7
Religion	Muslim	268	66.0
	Orthodox	91	22.4
	Others^a^	47	11.6
Ethnicity	Oromo	291	71.7
	Amhara	46	11.3
	SNNP^e^	41	10.1
	Others^b^	28	6.9
Educational status	Not educated	118	29.1
	1–8th grade	118	29.1
	9–12th grade	84	20.7
	College/above	86	21.2
Occupation	Farmer	161	39.7
	Gov’t employee	70	17.2
	Private employee	77	19.0
	Unemployed	87	21.4
	Others^d^	11	2.7
Residence area	Urban	203	50
	Rural	203	50
Relation to the patient	Parent	156	38.4
	Spouse	43	10.6
	Sibling	130	32.0
	Child	56	13.8
	Others^c^	21	5.2
Known diagnosed medical illness	Yes	18	4.4
	No	388	95.6

^a^Protestant, catholic, seven adventist, etc.

^b^Tigre, Gambella, Benshangul gumz, etc.

^c^Aunt, uncle, friends and neighbor

^d^Daily laborer, other informal jobs

^e^Southern nation nationalities and people like Gurage, Siltie, Keffa, Yem, etc.

Majority [248 (61.1%)] of the family caregivers provide care for male patients, one-third [136 (33.5%)] for patients who had primary education and only 61 (15.0%) for patients who had college or above educational level as shown in Table 2.

**Table 2:** Description of socio-demographic and clinical characteristics of patients (*N* = 406)

Variable	Category	*n*	%
Median age of the patient (IQR)		29.50/14.00	
Mean age onset of illness of patient (± SD)		26.10 ± 11.322	
Median patient duration of illness in years (IQR)		4.00/6.00	
Sex of the patient	Male	248	61.1
	Female	158	38.9
Employment status of patient	Unemployed	37	9.1
	Working full time	27	6.7
	Working part-time	97	23.9
	Stop working	245	60.3
Educational status of patient	Not educated	115	28.3
	1–8th grade	136	33.5
	9–12th grade	94	23.2
	College/above	61	15.0
Psychiatric diagnosis of the patient	Schizophrenia	210	51.7
	Other psychotic d/os^a^	39	9.6
	BAD	50	12.3
	MDD	80	19.7
	Other disorders^b^	27	6.7
Patients’ episodes of illness	Single	184	45.3
	2–4 episodes	138	34.0
	5 or more episodes	84	20.7
Patient history of psychiatric admission	Yes	130	32.0
	No	276	68.0
Patient ever use any type of substance in life	Yes	157	38.7
	No	249	61.3
Patient use of substance in the last 12 months	Yes	117	74.5
	No	40	25.5
Patient illness/CGI scale/	Normal	51	12.6
	Borderline ill	17	4.2
	Mildly ill	135	33.3
	Moderately ill	153	37.7
	Markedly ill	37	9.1
	Severely ill	13	3.2

*BAD* bipolar affective disorder, *MDD* major depressive disorder

^a^Brief psychotic, scizophreniform, delusional d/os, etc.

^b^PTSD, Anxiety d/os, Cognitive d/os, childhood d/os

As measured by the family burden interview schedule scale (FBIS), the mean total objective burden score was 23.00 ± 10.71 and the mean scores for each of the domains measured are illustrated in Table 3.

**Table 3:** Description of objective burden of caregivers according to FBIS domains

Variables	Mean	±SD	Range	
			Min	Max
FBIS (objective burden score)	23.00	10.716	0	48
Domains of burden				
A. Economic burden	6.60	2.982	0	12
B. Disruption of family routine activities	5.56	2.591	0	10
C. Disruption of family leisure overall	3.57	2.255	0	8
D. Disruption of family interaction overall	4.44	3.023	0	10
E. Effect on physical health of others	0.95	1.107	0	4
F. Effect on mental health of others	1.87	1.463	0	4

**Fig. 1:**
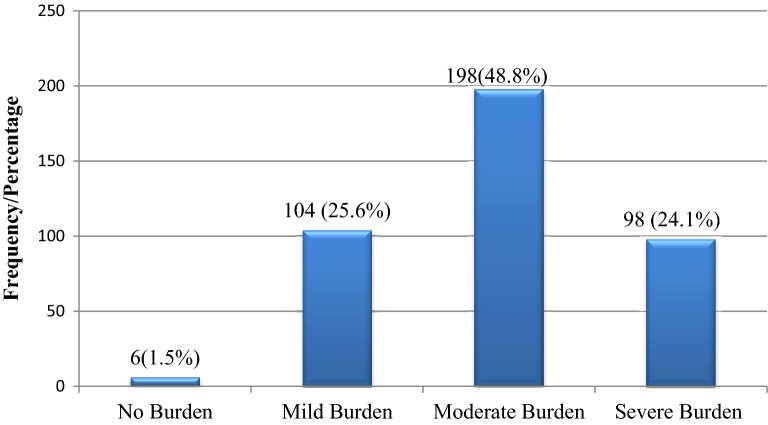
Caregivers level of total objective burden

**Fig. 2:**
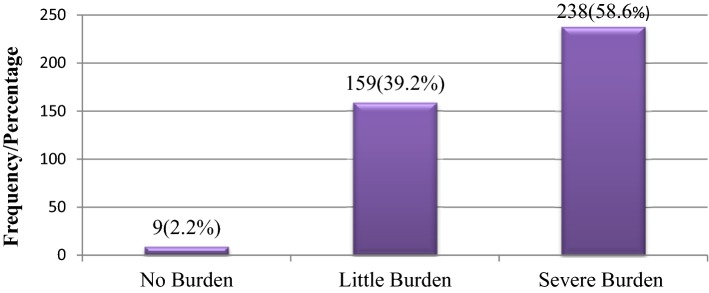
Caregivers global subjective burden level

In the global objective burden scale, 104 (25.6%) had mild burden, 198 (48.8%) moderate burden, and 98 (24.1%) had severe burden as illustrated in Fig. 1. Whereas on subjective burden scale, 238 (58.6%) reported severe burden, 159 (39.2%) reported moderate burden, and 9 (2.2%) caregivers reported no burden as shown in Fig. 2.

### Predictors of burden among caregiver

In the final model, among the many variables from simple linear regression (Table 4), included in the multivariable linear regression analysis, age of the caregiver (*β* = 0.18, *p* < 0.001), being female caregiver (*β* = 2.68, *p* < 0.01); being a spouse (*β* = 3.53, *p* < 0.01), sibling (*β* = 5.94, *p* < 0.01), child (*β* = 4.36, *p* < 0.001), and other relatives (*β* = 4.03, *p* < 0.05) to the patient; duration of stay with the patient per day (*β* = 0.74, *p* < 0.001), and perceived stigma score (*β* = 0.47, *p* < 0.001) were positively and significantly associated with burden of family caregiver. It was also shown that average monthly income of the caregiver (*β* = − 7.25, *p* < 0.001), caregivers who had no formal education (*β* = − 4.65, *p* < 0.05), and caregivers’ social support (*β* = − 0.77, *p* < 0.001) were negatively associated with burden of caregivers. Moreover, from patient-related factors, caring for patients who had primary educational level (*β* = 2.42, *p* < 0.05), providing care for patients who had history of substance use ever in their life (*β* = 1.52, *p* < 0.05), and caring for patients who were moderately ill (*β* = 2.69, *p* < 0.05) were associated positively with higher burden of caregiver, as illustrated in Table 5. The model also indicated that 70.4% of the variations in the outcome were explained by the variables in the model.

**Table 4:** Simple linear regression for burden among caregivers of PWMI (FBIS)

Variable	*R* ^2^	*B*	95% CI	*p* value
Age of caregiver	0.117	0.304	0.223 to 0.386	< 0.001
Duration of caregiver contact to patient within 24 h	0.351	1.954	1.695 to 2.214	< 0.001
Duration of care given by the caregiver	0.110	7.513	5.428 to 9.598	< 0.001
Caregiver’s sex				
Male	0.007	0		
Female		1.931	− 0.283 to 4.145	0.087
Marital status of caregiver				
Married caregiver^a^	0.020	0		
Single		− 2.795	− 5.125 to (− 0.465)	0.019
Others		5.589	− 1.904 to 13.082	0.143
Caregiver’s educational level				
(College or above)^a^	0.006	0		
Not educated		0.982	− 2.007 to 3.971	0.519
Primary education		2.321	− 0.668 to 5.310	0.128
Secondary education		0.925	− 2.309 to 4.159	0.574
Residence of the caregiver				
Urban	0.005	0		
Rural		− 1.488	− 3.576 to 0.601	0.162
Relationship to the patient				
Parent^a^	0.026	0		
Spouse		0.554	− 3.044 to 4.153	0.762
Siblings		− 2.338	− 5.643 to 0.867	0.150
Children		− 2.299	− 4.780 to 0.182	0.069
Other relatives		− 6.686	− 11.542 to (− 1.829)	0.007
Caregiver’s income in USD	0.220	− 0.106	− 0.126 to (− 0.087)	< 0.001
Social support of caregivers	0.285	− 1.687	− 1.949 to (− 1.426)	< 0.001
Perceived stigma of caregiver	0.397	0.990	0.871 to 1.109	< 0.001
Patient’s age	0.010	6.992	0.171 to 13.812	0.045
Duration of patient’s illness	0.102	7.121	5.055 to 9.188	< 0.001
Age of the patient at first onset of illness	0.001	− 0.034	− 0.127 to 0.058	0.470
Patient’s sex				
Male	0.001	0		
Female		− 0.497	− 2.644 to 1.649	0.649
Patient’s educational level				
College or more education^a^	0.008	0		
Not educated		− 2.418	− 5.753 to 0.918	0.155
Primary education		− 1.646	− 4.891 to 1.599	0.319
Secondary education		− 0.183	− 3.645 to 3.279	0.917
Diagnosis of the patient				
Schizophrenia^a^	0.057	0		
Other psychotic disorders		− 5.668	− 9.253 to (− 2.083)	0.002
BAD^b^		1.686	− 1.550 to 4.921	0.306
MDD^c^		− 4.827	− 7.528 to (− 2.125)	< 0.001
Other disorders^d^		− 3.403	− 7.607 to (− 0.801)	0.112
Episodes of illness				
Single episodes of illness^a^	0.051	0		
2–4 episodes		5.659	3.349 to 7.970	< 0.001
5 or more episodes		3.521	0.819 to 6.223	0.011
Patients history of psychiatric admission				
Yes	0.042	0		
No		− 4.407	− 6.903 to (− 2.511)	< 0.001
Patient’s history of substance use				
Yes	0.016	0		
No		− 2.783	− 4.916 to (− 0.651)	0.011
Severity of patient’s illness (CGIS)				
Normal (asymptomatic)^a^	0.114	0		
Borderline ill		2.941	− 2.653 to 8.535	0.302
Mildly ill		5.442	2.159 to 8725	0.001
Moderate ill		7.170	3.940 to 10.400	< 0.001
Marked ill		12.078	7.764 to 16.391	< 0.001
Severe ill		15.866	7.814 to 23.917	< 0.001
Extreme ill		19.294	10.673 to 27.915	< 0.001

^a^Reference group

^b^Bipolar affective disorder

^c^Major depressive disorder

^d^Anxiety disorders, post-traumatic stress disorders, neuro-cognitive disorders, etc.

**Table 5:** Final regression model for multivariable analysis for caregiver burden (FBIS)

	Unstandardized coefficients	*p* value	95.0% CI	
	*β*		Lower	Upper
Age of caregiver	0.189	< 0.001	0.101	0.277
Income of caregiver	− 7.257	< 0.001	− 9.482	− 5.032
Caregivers sex (female)	2.682	0.001	1.113	4.251
Caregiver who were				
Parent	0			
Spouse	3.537	0.009	0.870	6.205
Sibling	5.940	0.002	2.274	9.606
Son/daughter	4.364	< 0.001	2.073	6.655
Other relation (friends)	4.032	0.021	0.598	7.465
Duration of stay with a patient in 24 h	0.741	< 0.001	0.491	0.990
Social support to the caregiver (OSSS)	− 0.778	< 0.001	− 0.990	− 0.566
Perceived stigma by the caregivers (PSS)	0.472	< 0.001	0.363	0.581
Caregiver who had college/above^b^	0			
Caregiver who was illiterate	− 4.653	0.001	− 7.430	− 1.876
Attended patient college/above education^b^	0			
Primary education	2.424	0.030	0.235	4.614
Attended patient has no history of substance use in life	1.524	0.025	0.195	2.853
Severity of patient illness (CGIS^a^)				
Normal (no sign of illness currently)^b^	0			
Moderately ill	2.693	0.019	0.449	4.937

^a^Clinical global impression scale

^b^Reference group

## Discussion

This study showed that mental illness put marked effect on caregivers of PWMI. According to the global objective burden score, nearly three-fourth (72.9%) of caregivers experienced moderate to severe level of burden as a result of providing care to PWMI on the global objective burden score. Our finding was similar to studies from Iran [26] and New Delhi [27] which reported 73.6% and 75.1% for moderate/severe level of burden among caregivers, respectively. A similar study done in Butajira, Ethiopia, reported a lower level of caregiver burden, 63.3% [14]. However, it was lower than studies conducted in Chile (90.2%) [28] and Nigeria 85.3% [10] among caregiver of people with schizophrenia. The difference might be due to the fact that chronic course of and having some residual symptoms among schizophrenic patients has a greater burden on caregivers.

Furthermore, almost all caregivers (97.8%) report that they felt subjective burden because of their mentally ill family member, which was supported by a study conducted at Amanuel Mental Specialized Hospital, where 99.4% family caregivers reported subjective burden [15].

Our study found out that caregiving burden increases as age of the caregiver increases. This finding is in accordance with previous studies that suggested older caregivers experience higher levels of caregiving burden compared to their younger counterparts [11, 29, 30]. Older caregiver cannot deliver care accordingly and are worried about who will take over their caregiving roles when they are no longer alive [30]. Moreover, the young caregivers tend to have a better educational achievement, which possibly would contribute to a better socio-economic status or understanding of the condition [11]. They are also most likely to spend less time with mentally ill family members than their older counter parts [9].

This study also found out that the higher the caregivers’ income, the lower the caregiver burden. This was in agreement with previous studies [11, 31]. As previous studies stated that most of the patients were economically dependent on a family member [32] and higher degree of burden on the caregivers were associated with low income [33]. Since caregivers were not employed and not able to work mostly [34] because they quit working or reduce their working hours and spent their time in home to take care for their mentally ill relatives [32]. Above all, caregivers also lose their financial income for caring mentally ill patients, paying for expenses incurred directly by the patients through destruction of household materials because of the patients’ destructive and violent act, expenses for transportation to health institutions, and fees for treatment were the most complicated challenges for relatives in Ethiopian situation too.

The level of burden is higher among female caregivers and this was also corroborated by other studies [35]. This was attributed to the fact that female caregivers have more emotional, social, physical, financial, and relationship burden [8, 31]. Because of ongoing gender role differences, females were mostly considered to take the duty of providing direct care and exposed to various responsibilities such as mother, wage earner, household manager, and key emotional supporter [36–38]. In addition, most women are more often less advantaged in socio-economic status with less education and lower earnings to deal with the challenges of exhaustive caregiving role which increases their burden.

Caregivers’ relationship to the patient was another significant predictor of burden of care. That is being a spouse, sibling, son or daughter, and other relatives to the patient increases family caregivers’ burden as compared to being parents. This is consistent with previous study that caregivers who care for their child have more positive personal experiences [8]. Parent caregivers become more satisfied in providing care, and engage more positively and intimately as compared to other caregivers [39].

Caregivers who had no formal education had lower burden score as compared to those who had college and above educational level. This was in line with a study conducted in US which revealed that high-burden caregivers had fewer years of education [26]. This might be due to higher level of education gives a better insight of the complexity of providing care [40] and also educated caregivers might have fixed jobs and responsibilities. Other studies also found out that higher level of education linked to higher level of burden [15, 31].

However, caregivers who were providing care for patients who had primary educational level had high level of burden as compared to caring for patients who had college and/or above. This was supported by previous study [26] indicated that lower education level were associated with higher burden of caregivers. Greater burden may be due to a lesser earning capability and productivity of patients during remission period [26]. Also, educated patients may have better insight about their illness and seek help and treatment early, resulting in lesser caregivers’ burden [41].

Our study indicated that longer duration of contact hours to take care the patient per day was associated with higher caregiver burden. The more time the caregiver spent with the care recipient, they may have less time for themselves, which increases the caregivers’ experiencing of burden [30, 42]. Therefore, significantly higher burden scores were observed among caregivers who had spent long hours daily in providing care for the patients [43]. Contrary to this finding, a similar study stated that, due to the sociocultural sense of obligation to care for sick family members, caregivers who spend short caregiving hours per day may experience emotional burden [31].

Another important predictor of caregiver burden in our study was caregivers’ social support and those who have higher social support had lower caregiver burden and vice versa. This was supported by previous studies that caregivers who had less social support perceived higher burden [44] and higher financial, physical, emotional, and time-dependence burden were associated with poor social support [31]. Caregivers’ burden increased when there is poor informal support from others [42]. Furthermore, Magliano et al. also found that caregiver burden decreases among those who get support from others in the family system or community [45].

Our study also found out that caregivers’ perceived stigma associated positively with caregivers’ burden. This was in agreement with a study conducted at Amanuel Mental Specialized Hospital indicating that the higher the caregivers’ perceived stigma in caregiving, the more the caregiving burden [15]. Highly burdened caregivers feel inferior, useless, and ashamed because of their mentally ill relative and they had faced emotional disturbances [34].

In addition, we also found out that providing care for patients who had ever used substance in their life increases caregiver burden score as compared to their counterparts. This was supported by the previous study that found out caregivers of patients who use substances had higher emotional burden for themselves [46]. This may be due to the fact that mentally ill patients who abuse substance may either misuse caregiver resources or act offensively under the direct influence of psychoactive substances [32]. In addition, those patients who use substance might create physical, sexual, or emotional abuse/violenceand have higher degree of behavioral problems, poor family interaction, and financial resource drainage [47].

Moreover, there were significant positive associations between caregivers burden of care and patients’ severity of illness, while moderately ill patients had higher burden score as compared to less severely ill or nearly normal patient caregivers. Fujino and Okamura found out that patients’ severity of illness associated with caregiver burden [48]. Possibly this might be related to ‘that more severely ill patients became functionally impaired, thereby depend heavily on their caregivers [11] and disturbance in patient’s behavior and longtime illness which result in limiting time, energy, and attention of caregiver [48].

Our study investigates the extent and pattern of caregivers’ burden of care and its associated factors among caregivers of PWMI. Therefore, our study uses standardized, reliable, and valid data collection tools and incorporate several caregiver and patient factors to reduce the confounding effect and to reflect an actual representation of the caregiving burden in the area. It also includes caregivers of mentally ill people in general. However, our study also has certain limitations. Our study design was cross-sectional that does not show cause and effect relation, risk of biased responses such as social desirability bias by which caregivers either exaggerate or minimize their burden for some reason and using non-probability consecutive sampling method also might be considered as a limitation. Even though we use internationally validated instrument to measure caregiver burden, FBIS was not yet validated in Ethiopia.

## Conclusion

Caregivers were experiencing substantially high level of caregiving burden; nearly, three-fourth of patients report moderate/severe objective burden and more than 97% of participants feel burdened subjectively. A variety of characteristics of PWMI and their caregivers were associated with caregivers’ burden of care. Caregiver factors that were significantly associated with caregiver burden score were age, sex, income, educational status, relation to the patient, duration of contact hours with the patient per day, social support level, and perceived stigma. Moreover, patients’ educational status, history of ever substance use, and severity of illness were also significant factors associated with family caregivers’ burden of care. All these factors were accountable for 70.4% of the variations in family caregivers’ burden of care. In light of the findings from this study, policies and programs targeting mental health problems in the country should strengthen the advocacy for a strong mental health policy and health insurance scheme covering the mentally ill. It is also indispensable to integrate family caregiver actions and interventions into national mental health care plans. This helps to further effectively decrease the problem of mental health and provision of appropriate care. Clinicians are also expected to evaluate the caregivers with the purpose of awaking the increasing burden of care and promote interventions accordingly. In addition, mental health professionals educate people about mental health and the care that mentally ill people require to facilitate social support and reduce stigma. Empower families to share caregiving responsibilities and to lower the threshold for using respite care. Employers are also expected to be sensitized to support family caregivers by employing and encouraging PWMI in order to empower them. Mental health service delivery institutions are also recommended to provide at least fee-free mental health service. Individuals in the community are empowered and encouraged to support mentally ill patients and their caregivers in order to reduce stigma and provide good social support that reduces burden of care.

## Data Availability

The datasets used and analyzed during the current study are obtained from the corresponding author on reasonable request.

## Abbreviations

CGIS: Clinical Global Impression Scale
FBIS: family burden interview schedule
IQR: inter quartile range
IRB: Institutional Review Board
JUMH: Jimma University Medical Center
LMICs: low- and middle-income countries
OSSS: Oslo Social Support Scale
PWMI: people with mental illness
PSS: perceived stigma scale
SD: standard deviation
SPSS: Statistical Package for Social Science
